# The experimental methodology and comparators used for in vivo hernia mesh testing: a 10-year scoping review

**DOI:** 10.1007/s10029-020-02360-x

**Published:** 2021-01-12

**Authors:** T. Whitehead-Clarke, R. Karanjia, J. Banks, V. Beynon, S. Parker, D. Sanders, V. Mudera, A. Windsor, A. Kureshi

**Affiliations:** 1 Division of Surgery and Interventional Science, University College London, Charles Bell House, Foley Street, London, W1W 7TY UK; 2 Department of General Surgery, St George’s Hospital, London, UK; 3 Department of General Surgery, University College London Hospital, London, UK; 4 Deartment of General Surgery, North Devon NHS Healthcare Trust, London, UK; 5 Princess Grace Hospital, HCA Healthcare, London, UK

**Keywords:** Hernia, Mesh, Testing, Standardisation, In-vivo

## Abstract

**Purpose:**

Before being marketed, hernia mesh must undergo in vivo testing, which often includes biomechanical and histological assessment. Currently, there are no universal standards for this testing and methods vary greatly within the literature. A scoping review of relevant studies was undertaken to analyse the methodologies used for in vivo mesh testing.

**Methods:**

Medline and Embase databases were searched for relevant studies. 513 articles were identified and 231 duplicates excluded. 126 papers were included after abstract and full text review. The data extraction was undertaken using standardised forms.

**Results:**

Mesh is most commonly tested in rats (53%). 78% of studies involve the formation of a defect; in 52% of which the fascia is not opposed. The most common hernia models use mesh to bridge an acute defect (50%). Tensile strength testing is the commonest form of mechanical testing (63%). Testing strip widths and test speeds vary greatly (4–30 mm and 1.625–240 mm/min, respectively). There is little consensus on which units to use for tensile strength testing. Collagen is assessed for its abundance (54 studies) more than its alignment (18 studies). Alignment is not measured quantitatively. At least 21 histological scoring systems are used for in vivo mesh testing.

**Conclusions:**

The current practice of in vivo mesh testing lacks standardisation. There is significant inconsistency in every category of testing, both in methodology and comparators. We would call upon hernia organisations and materials testing institutions to discuss the need for a standardised approach to this field.

**Supplementary Information:**

The online version contains supplementary material available at 10.1007/s10029-020-02360-x.

## Introduction

Identifying an ideal mesh for hernia repair has been a subject of huge interest for general surgeons for decades. The first ‘modern’ polypropylene mesh was introduced by Usher in the late 1950′s [1], and its use to provide a tension free inguinal hernia repair was then popularised by Lichtenstein several years later [2]. In recent decades, mesh development has proceeded at an exponential rate as industry develops novel biomaterials with beneficial properties. To this day, hundreds of different mesh products have been tested and sold, with at least 70 different mesh products on the market [3].

Before hernia meshes can be marketed, developers must provide evidence of a product’s safety and efficacy. This evaluation is mostly undertaken in vivo: testing the mesh on live animal subjects and explanting the mesh for analysis. The purpose of this testing is to establish that a new mesh is at least equivalent, if not superior, to an established product. Regulation for this area began in 1977 with the US Food and Drug Administration (FDA) Bioresearch Monitoring Programme, but more recent guidelines such as those from the European Union (EU) in 1993 and then 2017 [4] have provided more detailed advice on the requirements for product approval. The FDA have developed a guideline outlining suitable testing methods for new mesh products [5], however, this does not provide detail on the technical aspects of experimentation nor the comparators that should be analysed. To this day there is no recognised gold standard for the methods used to test hernia meshes in vivo.

The lack of a recognised standard has led to significant variance in the literature. A recent study by Vogels et al. [6] sought to investigate this by reviewing the animal models used for hernia mesh testing. They reviewed 15 years of experimental mesh studies and demonstrated significant variety in the models used. Their work recommended that guidelines should be established to standardise such research. Articles from experts in the field such as Deeken et al. [7] have also described how variable methodology can be, and that data reported by different groups can be inconsistent. Our review investigates the field in more detail than previous studies by identifying the specific testing methods used, the parameters recorded and how they are measured.

Given recent controversy and media attention surrounding hernia mesh, as well as the most recent EU regulations [4], the field of in vivo mesh testing will become increasingly relevant in years to come. The data from in vivo studies are already commonly used to market mesh products and will be progressively used as justification to commence human trials.

Our work reviews in vivo mesh testing studies conducted between 2009 and 2019 and focuses on four specific areas:

- Animal specifications and mesh implant/explant techniques.
- Biomechanical testing of mesh/tissue samples.
- Histology: structural analysis of mesh/tissue samples.
- Histology: inflammatory cellular analysis of mesh/tissue samples.

Our review analyses the variability in these four areas of in vivo mesh testing. By doing so, it will help identify the need, if any, for standardisation in the field and the areas in which this is most urgently required.

## Methods

### Literature search

**Fig. 1:**
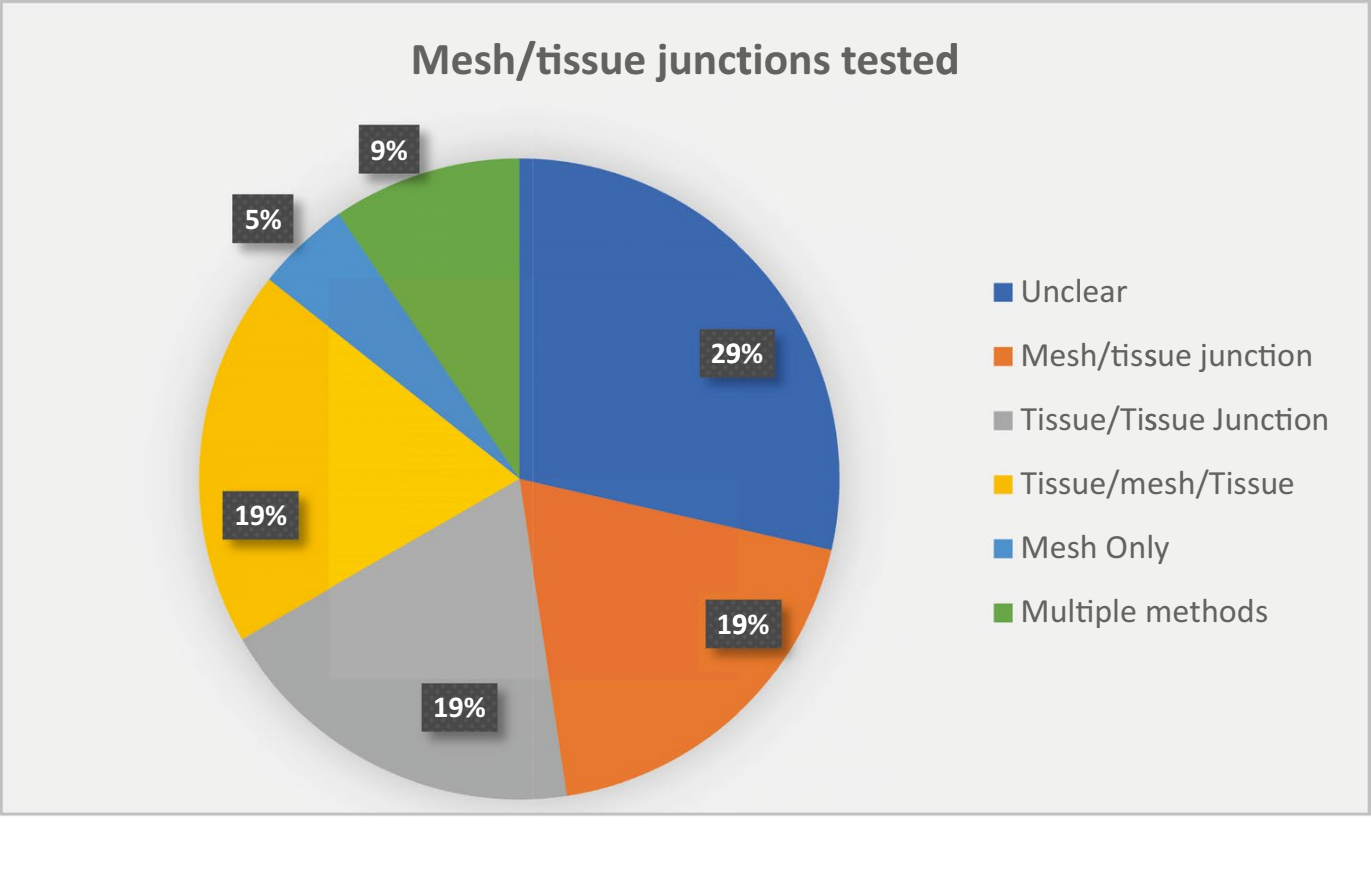
A chart showing the distribution of how testing strips were formed amongst 42 studies in which uniaxial tensile strength testing was undertaken

A search was conducted of the Medline and Embase databases using the OVID interface. The search was designed and conducted by a specialist science librarian from University College London (UCL). The search was designed to identify in vivo studies in which hernia mesh prostheses are implanted into animals and subsequently extracted for the purposes of testing. Articles in the English language were selected between January 2009 and October 2019. The specific search thread for both Embase and Medline can be found in our supplementary figures (figure 1).

### Article screening

The initial search produced 513 articles. 231 of these were duplicates and were excluded. Details of all remaining 282 papers were uploaded to Covidence online systematic review software (Covidence systematic review software, Veritas Health Innovation, Melbourne, Australia, www.covidence.org). Using this software, the remaining 282 articles underwent abstract review. Abstracts were assessed by four of the authors (TWC, RK, JB, VB). Each abstract was screened independently by two reviewers and was automatically included or excluded if there was consensus. In the case of disagreement, the final decision was referred to the lead author (TWC). Specific inclusion/ exclusion criteria were disseminated amongst the authors to standardise the process in which 144 articles were excluded. These criteria are provided below.

### Inclusion criteria

- Single arm studies and comparative studies that look to test the effectiveness and/or biocompatibility of surgical mesh.
- In vivo studies where the mesh and tissue sample has been explanted from the animal before testing.
- Studies looking at any type of mesh or mesh with a new coating. This includes synthetic, biological or composite meshes.
- Studies that examine mesh/ tissue interaction at the abdominal wall for Inflammatory, structural or biomechanical properties.
- Studies where the mesh is implanted in the abdominal wall.
- Studies published between the years January 2010 and October 2019 inclusive.
- Studies published in the English language

### Exclusion criteria

- Studies that compare or assess fixation technique.
- Studies where a primary subject of investigation is not mesh performance.
- Studies where a mesh or coating is exclusively assessed for adhesion formation or mesh shrinkage.
- Studies testing new pharmacological products.
- In-vitro studies.
- Studies where mesh is placed and assessed for femoral/obturator hernias.
- Studies where the mesh is placed around pelvic organs to test use in treatment for prolapse/incontinence.
- Studies where the mesh is used as part of a rectopexy procedure for prolapse surgery.

Following abstract review, the remaining 138 papers underwent full text review and data extraction. Papers were distributed equally between the four reviewing authors (TWC, RK, JB, VB) for simultaneous full text review and data extraction. 12 papers were excluded after full text review, due to an inappropriate study design (11), or the paper was not available (1). This left 126 papers to undergo data extraction. A full PRISMA diagram for article screening is available in our supplementary figures (Supplementary figure 2).

### Data extraction

The data extraction and entry were undertaken using a standardised online spreadsheet. Painstaking efforts were made to standardise data collection between authors. Authors met before the process began to discuss the data extraction, and how to appropriately record results. Further such meetings were held after five papers had been reviewed by each author, and at 2–3-week intervals thereafter until data collection was complete. The data collection forms were adapted during the process to reflect the results being collected.

A full list of variables that were measured from each study is outlined below. An initial protocol for the review has been published [8].

### Study/experimental data

- Primary variable assessed (e.g. type of mesh).
- Primary outcome assessed (e.g. tensile strength of mesh/tissue composite)
- Is the mesh new to market? (yes no/unclear).
- The use of a non-mesh control (yes/no).
- Animal species used.
- Animal subspecies used.
- Animal weight.
- Animal age.
- Number of animals used in the study.
- Defect shape (linear/2-dimensional).
- Defect depth (partial thickness/full thickness*).
- Defect size.
- Mesh size.
- Nature of defect (Chronic/Acute**).
- Plane of mesh placement.
- Defect closure (yes/no).
- Attachment of mesh (absorbable or non-absorbable/tacks or sutures).
- Times of mesh explantation (single/multiple and time in days).

### Mechanical testing

- Method of mechanical testing
- The units used to quantify material properties.
- Initial load on testing mechanism.
- Speed of testing mechanism.
- Width of testing samples.
- Cross-sectional area of testing samples.
- Structure of testing samples.
- Inter-clamp distance for tensile strength testing.
- Shape of testing strip for tensile strength testing (dog bone/Not dog bone).

### Histology—structural analysis

- The technique used to visualise collagen (e.g. staining).
- Differentiation of type 1 from type III collagen (yes/no).
- Aspects of collagen investigated (abundance/alignment/both).
- The scale used to measure the presence of collagen (quantitative/scored using quantitative assessment/scored using qualitative assessment/qualitative assessment).
- Other structural components assessed (e.g. tissue integration, fibrous encapsulation).
- The scale used to measure other structural components (quantitative/scored using quantitative assessment/scored using qualitative assessment/qualitative assessment).
- Use of scoring system (uncited/cited/no).

### Histology—inflammatory cellular analysis

- Types of cell assessed.
- The technique used to visualise each type of cell.
- The scale used to measure the presence of cells (quantitative/scored using quantitative assessment/scored using qualitative assessment/qualitative assessment).
- Use of scoring system (uncited/cited/no).

*Partial/full thickness defects do not/do include the peritoneum respectively.

**Acute defects are made and repaired during the same procedure. Chronic defects are made and allowed to mature before then being repaired.

## Results

126 studies were reviewed. The results are divided into four sections as described in our methods.

### Experimental technique

Rats represented the most common animal used for studies (53%) followed by pigs (24%) and rabbits (18%). Guinea pigs, dogs and sheep were also used in several studies. Subspecies included at least two rabbit species, three pig species and four rat species. 84% of studies provided details of animal weights, only 25% of studies provided details on animal ages. 78% of studies (98) involved the formation of a hernia defect. Table 1 below represents how hernia defects were formed and repaired.

**Table 1:** Summary of techniques used in defect formation and repair amongst the 98 studies in which a hernia defect was created

Type of defect formation and repair	Linear defect (%)	2D defect (e.g. square/circular) (%)
Acute defect, partial thickness, defect left open	2	21.4
Acute defect, partial thickness, fascia opposed	5.1	5.1
Acute defect, full thickness, defect left open	0	28.6
Acute defect, full thickness, fascia opposed	10.2	7.1
Chronic defect, partial thickness, defect left open	5.1	0
Chronic defect, partial thickness, fascia opposed	2	3.1
Chronic defect, full thickness, defect left open	6.1	2
Chronic defect, full thickness, fascia opposed	0	2
Total	30.6	69.4

The most common in vivo hernia models involve the formation of an acute defect in which the fascia is subsequently not closed (50% of studies with a defect). Two-dimensional defects (69.4%) such as square or circular ones are more than twice as common as linear defects (30.6%). Only 5% of studies with defects involved an appropriate physiological mimic of a human hernia repair (a partial thickness chronic defect). For those studies in which a defect was not formed, 75% of meshes were placed intraperitoneally.

The sizes of defects and mesh implants varied between studies. We used these measurements to assess the mesh/tissue overlap for studies where that information was available. Overall mean mesh/tissue overlap was 0.98 cm. This overlap was significantly larger for studies in which the fascia was opposed (1.6 cm) compared to studies using mesh to bridge a defect (0.51 cm) (Supplementary figure 2).

Mesh and tissue were explanted from animals at a variety of time points. 47% of studies explanted mesh and tissue from animals at one fixed time point (7–240 days, median 42 days). 53% of studies involved between two and six explantation time points (Table 2).

**Table 2:** The number of explantation points for each of the 126 studies

Number of explantation points	1	2	3	4	5	6
Number of studies (%)	47	26	16	6	3	2

### Mechanical testing

Of the 126 reviewed studies, 63 papers undertook mechanical testing of mesh/tissue samples. 67% of those studies used uniaxial tensile strength testing, making it the most frequently used technique. 16% and 10% of studies undertook T-Peel and ball-burst testing respectively. 3% of mechanical testing studies undertook biaxial tensile strength testing. Given the popularity of uniaxial tensile strength testing, we have used it as the focus for our mechanical testing analysis.

To undertake uniaxial tensile strength testing, strips are formed from the testing material and are pulled apart in order to assess various properties. In 29% of these studies, the formation of the testing strips was unclear. Figure 1 shows the formation of all testing strips used amongst the 42 studies that carried out uniaxial tensile strength testing. Explanatory diagrams of these testing strips can be found in our supplementary material (Supplementary figure 3).

The width and cross-sectional area of these testing strips was also analysed. 69% (29) of uniaxial tensile testing studies provided a width measurement for the testing strips used. Widths ranged from 4 to 30 mm (median 10 mm) and only three studies recorded a cross-sectional area. All tensile strength testing studies used rectangular strips—apart from four which chose to use “dog bone” shaped strips. Testing speeds were documented in 86% of studies, which ranged from 3 to 240 mm/min (median 25.2 mm/min).

Amongst the uniaxial tensile testing studies reviewed, different material properties were measured and different units used. For each study, every measurement that was taken and its associated unit of scale was recorded. Table 3 summarises the number of studies in which each measurement was used.

**Table 3:**
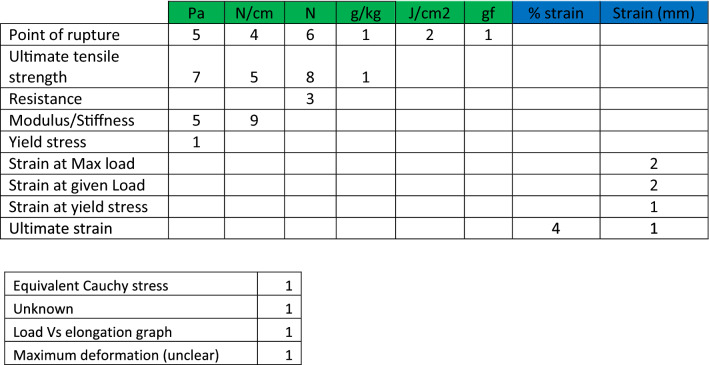
Number of studies in which certain measurements and units are used to assess a range of material properties

One study may contribute more than one measurement. Green columns represent stress measurements, and blue columns represent strain measurements. 4 outliers are described in the smaller table

### Histology—structural analysis

All histopathological structural factors encountered throughout the review were recorded. For sake of analysis, we have only included factors encountered in 10 or more studies.

**Fig. 2:**
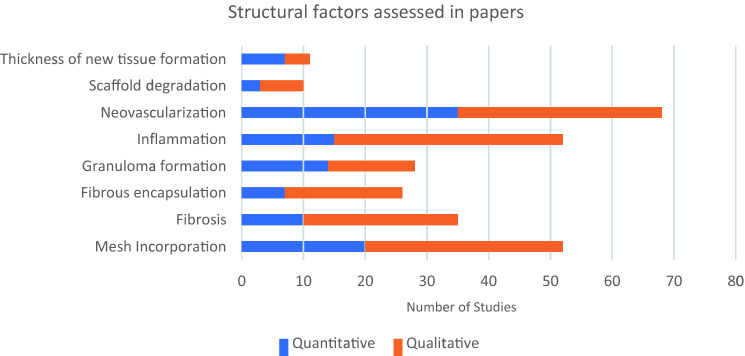
A graph showing the number of studies in which each structural factor was assessed. Blue represents studies which used quantitative methods, and orange qualitative methods (colour figure online)

Histological structural factors most frequently assessed include neovascularisation, inflammation and mesh incorporation. Figure 2 demonstrates all the structural factors that were encountered in the review (when *n* ≥ 10). Data has been divided into those factors that underwent either qualitative or quantitative analysis; either category can include the use of a scoring system.

**Fig. 3:**
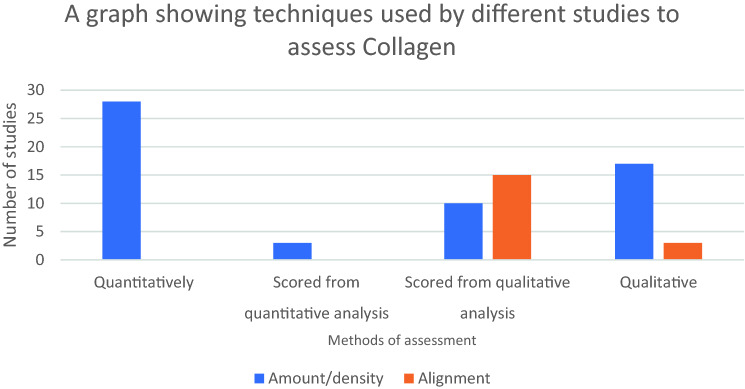
A graph showing the number of studies to assess collagen in terms of abundance or alignment. Assessment can be made purely qualitatively or quantitatively or with the use of a scoring system

The presence of collagen was assessed in 47% (59/126) of all studies. 69% of those studies measured its abundance, 7% measured its alignment or organisation, and 24% measured both. Collagen abundance was most often measured quantitatively; however, its alignment or organisation was exclusively measured qualitatively (Fig. 3). Collagen was vizualised most frequently through staining and light microscopy (71%), but also polarising microscopy (17%) and immunostaining (12%).

### Histology—inflammatory cellular analysis

**Fig. 4:**
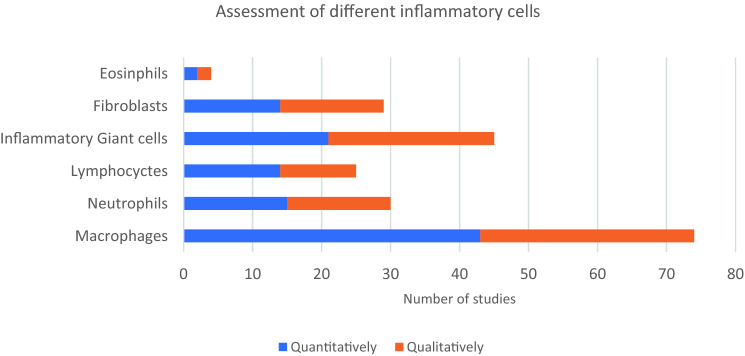
A graph showing the number of studies in which each inflammatory cell was assessed. Blue represents studies which used quantitative methods, and orange for qualitative methods (colour figure online)

67% (85/126) studies undertook an assessment of inflammatory cells. The most commonly analysed cell types were macrophages and giant cells. Macrophages were visualised microscopically with simple stains such as haematoxylin and eosin (82%) or with immunostaining (18%). Studies used both qualitative and quantitative assessment to identify inflammatory cells (Fig. 4).

For both the structural and inflammatory cellular analysis, scoring systems were frequently used to summarise results. 57 papers used a scoring system for structure and 36 for cellularity, but there was significant overlap between the two. 35 different papers used scoring systems that were either uncited or created by the authors. Within those papers that used a cited or established scoring system, 21 different scoring systems were identified.

## Discussion

Hernia meshes have been tested in vivo for decades; dating back to the 1950s, 60 s and 70 s when the testing of products such as stainless steel and silver meshes [9] was commonly conducted on dogs [10] but also pigs, rats and rabbits [11–13]. Whilst a few studies have examined hernia meshes in human and animal models simultaneously [14] the question of how in vivo experiments translate to patient outcomes is still not clear. Part of this issue may lie in the animal species used for in vivo studies and how they relate to human anatomy and physiology.

Our review indicates that rats (53%) are the animal most frequently used for in vivo studies—a finding identical to that of Vogels et al. [6] in 2017 (53.3%). This is unsurprising given its obvious financial advantages but provides poor physiological and anatomical compatibility. Not only are there significant differences in terms of size and fascial thickness, but rats also possess a superior ability to heal [15]. Experts in the field have pointed to porcine models as the most physiologically appropriate option [7], especially for mechanical testing. Given that only 24% of studies are using porcine models, much research may be devoid of this physiological accuracy.

84% of the reviewed studies documented a weight for the animals used- usually from the beginning of each study. Whilst such details may appear trivial, any growth of animals throughout a study can have a significant effect upon results. When Cerise et al. conducted one of the early mesh testing studies in 1975 [16], they found that rats doubled in size during the study. This growth mirrored a similar increase in the bursting strength of the abdominal wall, which also doubled. The use of adult Vs juvenile animals was not measured in this review, however perhaps the size of each animal is not as important as the *change* in that size.

In the studies reviewed, a variety of techniques are reported for the formation and repair of hernia defects—most of which are dissimilar to defects in human hernia repair. Experimental defects are most likely to be two-dimensional, with a mesh used to bridge un-opposed fascia (50% of studies with defects). A “bridged” repair was defined as any hernia repair where the mesh was placed in the space between the two fascial layers and these fascial layers were not closed. We did not differentiate between onlay, inlay and intraperitoneal bridging, and our description does not therefore adhere strictly to the recently published ICAP guidelines [17]. Given that fascial opposition is recommended for ventral hernia repair [18], we must question whether such bridged models are best placed to assess hernia mesh. Theoretically, one model demonstrates a mesh’s ability to support a repair, whilst the other assesses de novo fascial synthesis. Most human hernial defects are also partial thickness in nature (no peritoneal defect) and are repaired after the hernia has been present for some time (i.e. a chronic hernia). When looking for studies which fit these characteristics, few can be found. Only 5% of studies involving the repair of a defect used “mature” partial thickness defects where fascia was opposed upon repair. It is also accepted best practice that hernia mesh should overlap fascial closure. The Danish hernia registry recommends an overlap of at least 1 cm for small (< 2 cm) defects and 3 cm for larger (2–6 cm) defects [18]. Within our review, the data could be gathered for 39 studies that used the most common experimental model (bridged defects). Of the 39 studies, only 7 involved an overlap of 1 cm or more and 10 did not involve any overlap at all. Our data support the hypothesis that in vivo studies poorly mimic the physiology and anatomy of human hernia repair.

The decision of when to explant tissue should depend upon the nature of the subsequent analysis. Studies in our review were split evenly between those with multiple explantation time points (53%) and those with a singular explantation point (47%). Studies with a singular explantation time varied *greatly* between 7 and 240 days (median 42). Healing of the abdominal wall happens in a number of stages, typically taking several months to reach completion [19] and will appear histologically different throughout that time. A better understanding is required of the healing milestones in animal models to appreciate any histological analysis made at a single time point. Not only this, but If samples are analysed mechanically before full maturation of the healing process, then testing relies too heavily on mesh strength and fixation, rather that fascial healing.

Uniaxial tensile strength testing is the technique most frequently used for mechanical testing. Interestingly, some of the earliest mechanical testing of hernia mesh performed in vivo was in the form of burst strength testing in the 1970’s [16, 20]. Despite this being an arguably more physiological test, it constitutes less than 10% of the testing in our data. Whilst there are several studies looking at uniaxial tensile strength of human rectus sheath [21, 22], there are few that have examined its burst strength [23]. It may be this emphasis on uniaxial testing in human tissue (and its ease of practice) that has fuelled its popularity in mesh testing.

Uniaxial tensile strength testing uses tissue/mesh samples cut into strips, that are pulled apart by testing apparatus. The constituent parts of these testing strips can range from a simple mesh/tissue interface [24] to two sides of healed fascia and underlying mesh being pulled apart [25]. Strips of different designs will inevitably test different aspects of the tensile strength of the sample. Such factors may include the strength of integration between mesh and tissue, the strength of the mesh itself, the strength of healed fascia or indeed a mixture of all three. Such variation in practice leaves us to question not only what *is* being tested, but also what *should* be tested? Areas such as mesh/tissue integration can be assessed through both tensile strength testing and T-Peel testing. The establishment of one of these two as the methodology of choice provides a basic example of the possible standardisation in the field.

Tensile strength testing is performed at a set speed (mm/min), and it is well understood that polymers display different tensile strength characteristics at different testing speeds [26]. To combat phenomena, such as this, international testing standards have been developed by groups such as The International organisation for standardisation (ISO). ISO have published a range of standards for materials testing, including the tensile strength testing of plastics [27]. These standards; however, are designed for extrusion or machined plastics rather than polymer mesh. ASTM International (previously the American Society for Testing and Materials) have published guidelines for the testing of “non-rigid” plastics, and advised for a test speed of either 50 or 500 mm/min [28]. These standards are only applicable for “standard dumbbell shaped specimens” and it is unclear whether this includes polymer meshes. The extensive ex vivo work done by Deeken et al. used a testing rate of 25 mm/min [29, 30] without identifying specific guidance. Given that some hernia mesh resembles a woven/knitted fabric, other ex vivo studies have tested mesh at a rate of 50 mm/min—citing guidelines used for fabric testing [31]. Our review identified a significant range of testing speeds (1.62–240 mm/min) which is unsurprising given the apparent gap in the guidance, particularly with regards to mesh/tissue composites. Developing any such guidance would be difficult given the mixture of gentle and more harsh movements of the human abdominal wall. In this case perhaps standardisation between studies would be more useful than physiological accuracy.

In our review, the widths of the testing strips ranged from 4 to 30 mm. Both ASTM international and ISO recommend that plastic strips for tensile strength testing should be a regulated width and shape. The ISO standards recommend that testing strips should be a specific shape with the narrow portion measuring anywhere from 10 to 2 mm depending upon the overall size of the sample [27]. The ASTM standards for non-rigid plastics recommend a testing strip width of either 19 mm or 6 mm [28]. Both these standards also recommend a “dog bone” shape for each testing strip—something not recommended by ISO for testing fabrics [32], and only adopted by four of the studies we reviewed.

The ISO standards for the tensile strength testing of plastics provide detailed definitions for mechanical terms such as *stress at break* and *elasticity modulus* as well as the appropriate units for each measurement [27]. Stress measurements must be represented in MPa (N/mm^2^), and strain should be measured as a dimensionless ratio or percentage. Studies within this review use a number of different units including N/mm^2^, N/mm and also solely Newtons. Whilst these units are convertible given the cross-sectional area of a testing strip, only 3 articles provided this data. The variation in units (N/mm^2^ vs N/mm) stems from the way plastic meshes are tested in vivo and ex vivo respectively. Many ex vivo studies display their results in terms off N/mm—treating hernia mesh as essentially a 2D structure [7]. When a mesh/tissue interface is tested, the thickness and therefore cross-sectional area of the sample must be accounted for. Failure to do so has the potential to breed inconsistency.

Histopathological assessment of mesh/tissue samples is commonplace amongst in vivo studies and the lay down of collagen is often used as a surrogate marker for healing. Amongst our data, collagen appears to be measured far more frequently by its abundance (43%) than by its alignment or organisation (14%). Coupled with the fact that it is measured purely qualitatively, it could be assumed that collagen alignment is considered a less relevant factor to analyse. There is evidence from the literature as early as 1985 that researchers were interested in the alignment of collagen whilst testing the efficacy of hernia mesh [33]. There is also good evidence that the physical attributes of the rectus sheath (namely its anisotropy) are linked to the alignment of its collagen fibres [22], which form either an oblique pattern or a transverse pattern dependent upon the layer of aponeurosis [34, 35]. Studies have also shown that disorganised scar tissue in the rectus sheath lacks the strength of organised native tissue [21]. If collagen alignment is indeed an important factor in the strength of wound healing, then measuring this quantitatively might become an important factor for future research. Work from our group has already displayed how collagen alignment in vitro [36] and tissue collagen fibril analysis [37] can be conducted quantitatively through electron microscopy.

Our review divides histopathological assessments of mesh into quantitative and qualitative measurements as well as those that use a scoring system. A significant number of studies reviewed (particularly structural assessment) present data purely qualitatively—providing only a brief descriptive summary of results and no objective analysis. Cellular inflammatory data are measured more quantitatively, which is unsurprising given the relative ease of quantification.

A number of the histopathological studies across this review gather and present their data in the form of a scoring system. Our review identified 57 different papers which used a scoring system to assess structure, and 36 which assessed cellular activity. 35 papers were identified which used their own unique scoring systems or failed to cite a previously recognised one. Within the papers that cited recognised scores, we identified 21 separate systems. Whilst most of those scoring systems are taken from other in vivo mesh studies, a number are from very different experiments including looking at the effect of mesh upon a rabbit vaginal model [38] or looking at inflammation in murine bowel anastomosis [39]. We identified only one study which cited an internationally recognised scoring system [40].

This study has its limitations. The data has only been taken from the last 10 years and studies before 2010 may reveal significant differences. Furthermore, only studies analysing mechanical, structural and cellular assessment were included. Looking at other factors such as adhesions or mesh shrinkage may provide significantly higher rates of standardisation. Initial inclusion/exclusion of papers was conducted using abstracts, which may have introduced inaccuracies. Finally, this study does not represent a rigorous systematic review of the subject and rather represents a summary of the literature to assist further studies. Whilst a protocol was designed, new factors were included in the study if they were of significant relevance.

## Conclusions

In vivo mesh testing literature from the last decade varies in its methodology and comparators. The techniques used for mesh implantation and defect formation vary significantly and often represent a model unrepresentative of human hernia surgery. Mechanical testing requires closer adherence to current standards or production of new standards all together. Histopathological scoring systems vary throughout the literature and would benefit from standardisation. Whilst individual studies may be scientifically sound, harmonisation of techniques and comparators across the field would create a more robust data set for systematic review or meta-analysis. Our group has begun work on a standardised in vitro model for mesh testing to standardise the testing substrate; however, we would invite testing agencies as well as the hernia community to discuss standardisation of testing methods and how this may be achieved.

## Supplementary Information

Below is the link to the electronic supplementary material.

Supplementary file1 (DOCX 358 KB)

## Data Availability

Not applicable.

## References

[1] Usher FC (1959). A new plastic prosthesis for repairing tissue defects of the chest and abdominal wall. Am J Surg.

[2] Lichtenstein IL, Shulman AG, Amid PK, Montllor MM (1989). The tension-free hernioplasty. Am J Surg.

[3] Baylon K, Rodriguez-Camarillo P, Elias-Zuniga A, Diaz-Elizondo JA, Gilkerson R, Lozano K (2017). Past, present and future of surgical meshes: a review. Membranes (Basel).

[4] Regulation (EU) 2017/745 of the European Parliament and of the Council of 5 April 2017 on medical devices, amending Directive 2001/83/EC, Regulation (EC) No 178/2002 and Regulation (EC) No 1223/2009 and repealing Council Directives 90/385/EEC and 93/42/EEC (Text with EEA relevance)Text with EEA relevance. (2017). Accessed 28/11/2019 2019

[5] United States Food and Drug Administration 1999—Guidance for the Preparation of a Premarket Notification Application for a Surgical Mesh—Guidance for Industry and/or for FDA Reviewers/Staff and/or Compliance https://www.fda.gov/regulatory-information/search-fda-guidance-documents/guidance-preparation-premarket-notification-application-surgical-mesh-guidance-industry-andor-fda. Accessed Jan 2020

[6] Vogels RRM, Kaufmann R, van den Hil LCL, van Steensel S, Schreinemacher MHF, Lange JF, Bouvy ND (2017). Critical overview of all available animal models for abdominal wall hernia research. Hernia.

[7] Deeken CR, Lake SP (2017). Mechanical properties of the abdominal wall and biomaterials utilized for hernia repair. J MechBehav Biomed Mater.

[8] Whitehead-Clarke TPS, Parisi V, Windsor A, Mudera V, Kureshi A (2020). The experimental methodology and comparators used for in vivo hernia mesh testing: a protocol for scoping review. Int J Sci Res Methodl.

[9] DeBord JR (1998). The historical development of prosthetics in hernia surgery. SurgClin N Am.

[10] Narat JK, Khedroo LG (1952). Repair of abdominal wall defects with fortisan fabric; experimental study. Ann Surg.

[11] Bollwahn W, Messow C, Zschocke G (1964). On the closure of large wounds and the retention of Perlon mesh in umbilical hernias of pigs. DtschTierarztlWochenschr.

[12] Rayner CR (1974). Repair of full-thickness defects of the abdominal wall in rats avoiding visceral adhesions. Br J PlastSurg.

[13] Elliott MP, Juler GL (1979). Comparison of Marlex mesh and microporousteflon sheets when used for hernia repair in the experimental animal. Am J Surg.

[14] van den Hil LCL, Vogels RRM, van Barneveld KWY, Gijbels MJJ, Peutz-Kootstra CJ, Cleutjens JPM, Schreinemacher MHF, Bouvy ND (2018). Comparability of histological outcomes in rats and humans in a hernia model. J Surg Res.

[15] Weber B, Lackner I, Haffner-Luntzer M, Palmer A, Pressmar J, Scharffetter-Kochanek K, Knöll B, Schrezenemeier H, Relja B, Kalbitz M (2019). Modeling trauma in rats: similarities to humans and potential pitfalls to consider. J Translat Med.

[16] Cerise EJ, Busuttil RW, Craighead CC, Ogden WW (1975). The use of Mersilene mesh in repair of abdominal wall hernias: a clinical and experimental study. Ann Surg.

[17] Parker SG, Halligan S, Liang MK, Muysoms FE, Adrales GL, Boutall A, de Beaux AC, Dietz UA, Divino CM, Hawn MT, Heniford TB, Hong JP, Ibrahim N, Itani KMF, Jorgensen LN, Montgomery A, Morales-Conde S, Renard Y, Sanders DL, Smart NJ, Torkington JJ, Windsor ACJ (2020). International classification of abdominal wall planes (ICAP) to describe mesh insertion for ventral hernia repair. Br J Surg.

[18] Henriksen NA, Bisgaard T, Andersen HF, Jørgensen LN, Helgstrand F (2018). [Surgical treatment algorithm for ventral hernias]. Ugeskr Laeger.

[19] Chintamani (2018). Editorial: ten commandments of safe and optimum abdominal wall closure. Indian J Surg.

[20] Arnaud JP, Eloy R, Adloff M, Grenier JF (1977). Critical evaluation of prosthetic materials in repair of abdominal wall hernias: new criteria of tolerance and resistance. Am J Surg.

[21] Hollinsky C, Sandberg S (2007). Measurement of the tensile strength of the ventral abdominal wall in comparison with scar tissue. ClinBiomech (Bristol, Avon).

[22] Grassel D, Prescher A, Fitzek S, Keyserlingk DG, Axer H (2005). Anisotropy of human lineaalba: a biomechanical study. J Surg Res.

[23] Rath AM, Zhang J, Chevrel JP (1997). The sheath of the rectus abdominis muscle: an anatomical and biomechanical study. Hernia.

[24] Ponce Leon F, Manso JEF, Abud VL, Nogueira W, Silva PC, Martinez R (2018). Sublay repair results in superior mesh incorporation and histological fibrogenesis in comparison to onlay and primary suture in an experimental rat model. Hernia.

[25] Cavallo JA, Greco SC, Liu J, Frisella MM, Deeken CR, Matthews BD (2015). Remodeling characteristics and biomechanical properties of a crosslinked versus a non-crosslinked porcine dermis scaffolds in a porcine model of ventral hernia repair. Hernia.

[26] Siviour C, Jordan J (2016). High strain rate mechanics of polymers: a review. J DynBehav Mater.

[27] ISO/BSI (2019) BS EN ISO 527–1:2019 Plastics. Determination of tensile properties. General principles. https://bsol.bsigroup.com/Bibliographic/BibliographicInfoData/000000000030377399. Accessed July 2020

[28] ASTM Standard Test Method for tensile properties of plastics 1. 10.1520/d0638-14

[29] Deeken CR, Abdo MS, Frisella MM, Matthews BD (2011). Physicomechanical evaluation of polypropylene, polyester, and polytetrafluoroethylene meshes for inguinal hernia repair. J Am CollSurg.

[30] Deeken CR, Abdo MS, Frisella MM, Matthews BD (2011). Physicomechanical evaluation of absorbable and nonabsorbable barrier composite meshes for laparoscopic ventral hernia repair. SurgEndosc.

[31] Pott PP, Schwarz MLR, Gundling R, Nowak K, Hohenberger P, Roessner ED (2012). Mechanical properties of mesh materials used for hernia repair and soft tissue augmentation. PLoS ONE.

[32] ISO/BSI (2013) BS EN ISO 13934–1:2013 Textiles. Tensile properties of fabrics. Determination of maximum force and elongation at maximum force using the strip method. https://bsol.bsigroup.com/Bibliographic/BibliographicInfoData/000000000030254791. Accessed July 2020

[33] Cameron AE, Taylor DE (1985). Carbon-fibre versus Marlex mesh in the repair of experimental abdominal wall defects in rats. Br J Surg.

[34] Axer H, von Keyserlingk DG, Prescher A (2001). Collagen fibers in lineaalba and rectus sheaths. J Surg Res.

[35] Axer H, Keyserlingk DG, Prescher A (2001). Collagen fibers in linea alba and rectus sheaths. I. General scheme and morphological aspects. J Surg Res.

[36] Mukhey D, Phillips JB, Daniels JT, Kureshi AK (2018). Controlling human corneal stromal stem cell contraction to mediate rapid cell and matrix organization of real architecture for 3-dimensional tissue equivalents. ActaBiomater.

[37] Kureshi A, Vaiude P, Nazhat SN, Petrie A, Brown RA (2008). Matrix mechanical properties of transversalis fascia in inguinal herniation as a model for tissue expansion. J Biomech.

[38] Huffaker RK, Muir TW, Rao A, Baumann SS, Kuehl TJ, Pierce LM (2008). Histologic response of porcine collagen-coated and uncoated polypropylene grafts in a rabbit vagina model. Am J ObstetGynecol.

[39] Vizzotto Junior AO, Noronha Ld, Scheffel DLH, Campos ACL (2003). Influência da cisplatinaadministrada no pré e no pós-operatóriosobre a cicatrização de anastomoses colônicasemratos. JornalBrasileiro de Patologia e Medicina Laboratorial.

[40] Melman L, Jenkins ED, Hamilton NA, Bender LC, Brodt MD, Deeken CR, Greco SC, Frisella MM, Matthews BD (2011). Histologic and biomechanical evaluation of a novel macroporous polytetrafluoroethylene knit mesh compared to lightweight and heavyweight polypropylene mesh in a porcine model of ventral incisional hernia repair. Hernia.

