# Supplementary material for: The experimental methodology and comparators used for in vivo hernia mesh testing: a 10-year scoping review
Source: Hernia. 2021 Jan 12;26(1):297–307. doi: 10.1007/s10029-020-02360-x (PMC8881265; doi:10.1007/s10029-020-02360-x)
Supplement: Supplementary file 1 — Supplementary file1 (DOCX 358 KB) [file 10029_2020_2360_MOESM1_ESM.docx]

***Supplementary Figures (Fig1-3):***

***Fig1 –*** *Literature search threads used for Embase and Medline.*

***Medline***

| 1. hernia, abdominal/ or hernia, inguinal/ or exp hernia, ventral/ or incisional hernia/ |  |
| --- | --- |
| 2. ((abdominal or incisional or Ventral or inguinal or postoperative or parastomal or umbilical) adj3 hernia*).tw. |  |
| 3. 1 or 2 |  |
| 4. Surgical Mesh/ |  |
| 5. mesh*.tw. |  |
| 6. 4 or 5 |  |
| 7. Materials Testing/ |  |
| 8. test*.tw. |  |
| 9. assess*.tw. |  |
| 10. compar*.tw. |  |
| 11. measur*.tw. |  |
| 12. or/7-11 |  |
| 13. 3 and 6 and 12 |  |
| 14. limit 13 to animals |  |
| 15. limit 14 to (english language and yr="2009 -Current") |  |

***Embase***

| 1. abdominal wall hernia/ or inguinal hernia/ or parastomal hernia/ or spigelian hernia/ or umbilical hernia/ |  |
| --- | --- |
| 2. incisional hernia/ |  |
| 3. ((abdominal or incisional or ventral or inguinal or postoperative or post-operative or parastomal or umbilical) adj3 hernia*).tw. |  |
| 4. 1 or 2 or 3 |  |
| 5. surgical mesh/ or mesh plug/ or nonabsorbable mesh/ or titanium mesh/ or transabdominal mesh/ |  |
| 6. mesh*.tw. |  |
| 7. 5 or 6 |  |
| 8. materials testing/ |  |
| 9. test*.tw. |  |
| 10. assess*.tw. |  |
| 11. compar*.tw. |  |
| 12. measur*.tw. |  |
| 13. 8 or 9 or 10 or 11 or 12 |  |
| 14. 4 and 7 and 13 |  |
| 15. limit 14 to animals |  |
| 16. limit 15 to (english language and yr="2009 -Current") |  |

***Fig 2:*** *PRISMA flow diagram representing article screening process.*


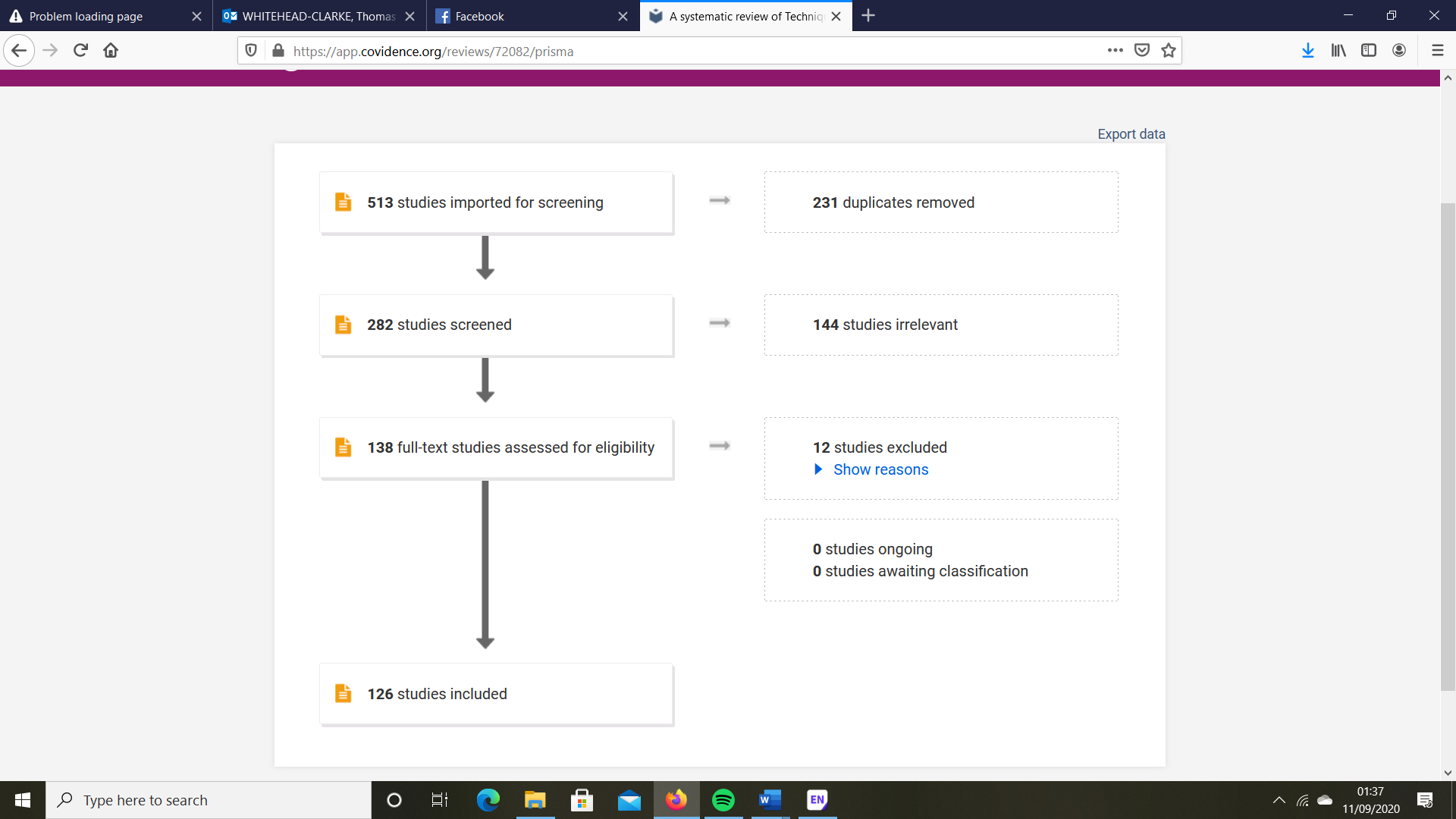


***Fig 3:*** *A graph assessing the mesh/tissue overlap in studies where a defect was made. Blue bars represent opposed repairs where orange bars represent bridged repairs.*

***Fig 4****: Pictorial representations of mesh/tissue testing strips and how they are described.*

Mesh/Tissue interface

Tissue/Tissue Interface with mesh

Tissue/Mesh/Tissue (generally for bridged defects)
